# The Use of Metallic Nanoparticles in Wound Healing: New Perspectives

**DOI:** 10.3390/ijms232315376

**Published:** 2022-12-06

**Authors:** Carolini Mendes, Anand Thirupathi, Maria E. A. B. Corrêa, Yaodong Gu, Paulo C. L. Silveira

**Affiliations:** 1 Faculty of Sports Science, Ningbo University, Ningbo 315211, China; 2 Laboratory of Experimental Phisiopatology, Program of Postgraduate in Science of Health, Universidade do Extremo Sul Catarinense, Criciúma 88806-000, Brazil

**Keywords:** wound healing, metallic nanoparticles, drug delivery, nanocomposites, scaffolds, green synthesis

## Abstract

Chronic wounds represent a challenge for the health area, as they directly impact patients’ quality of life and represent a threat to public health and the global economy due to their high cost of treatment. Alternative strategies must be developed for cost-effective and targeted treatment. In this scenario, the emerging field of nanobiotechnology may provide an alternative platform to develop new therapeutic agents for the chronic wound healing process. This manuscript aims to demonstrate that the application of metallic nanoparticles (gold, silver, copper, and zinc oxide) opened a new chapter in the treatment of wounds, as they have different properties such as drug delivery, antimicrobial activity, and healing acceleration. Furthermore, metallic nanoparticles (NPs) produced through green synthesis ensure less toxicity in biological tissues, and greater safety of applicability, other than adding the effects of NPs with those of extracts.

## 1. Introduction

Over the last few years, there has been a significant increase in patients with wounds around the world, especially pressure, venous and diabetic injuries, as they often occur in growing populations, such as the elderly, obese, and diabetics [1]. However, this problem, in addition to the elderly, affects the population in general and constitutes a public health problem, due to high treatment rates and costs [2]. Chronic wounds are widely regarded as a silent epidemic that endangers global health and the world’s economy. These wounds that do not heal with standard therapy in an orderly and timely manner cause further deterioration in these patients’ quality of life and increase the burden on the healthcare system over an extended period [3].

In chronic wounds, such as diabetic wounds, the inflammatory response, which is initially so vital, has a tendency to become exacerbated. Increased inflammatory cell infiltration, pro-inflammatory cytokine secretion, reactive oxygen species formation, and proteolytic enzyme synthesis are paired with decreased secretion of tissue inhibitors of metalloproteinases [4,5].

Increased expression of cytokines, such as tumor necrosis factor (TNF)-, interleukin (IL)-1, and IL-6, increases the manufacture of various matrix metalloproteinases, that, in excess, not only destroy the extracellular matrix (ECM) but also inactivate growth factors [4,6,7,8]. As a result, the chronic wound environment includes persistent matrix breakdown, decreased growth factor bioavailability, and enhanced fibroblast senescence, all of which work together to limit cell proliferation, angiogenesis, and tissue repair.

Alternative solutions for cost-effective and focused treatment must be explored. According to global research of the wound care management market, patients with chronic wounds are receiving better care as a result of newly developed solutions that are secure, effective, and beneficial for faster recovery [9]. In this context, the burgeoning field of nanobiotechnology could provide an alternate platform for developing new therapeutic medicines for wound infections.

The purpose of this paper is to show how nanotechnology, through the use of nanomaterials, has introduced a new era in wound healing, providing methods for faster healing and displaying differentiated capabilities as bactericidal agents [10,11].

Metallic nanoparticles have been considered for clinical application in this field because of their economical rate, high surface-to-volume ratio, stability, and safeness. Important applications of nanotechnology are being employed in healthcare and basic knowledge of the interaction of nanomaterials with cells and its biological effects is only being started [12,13].

There are three main criteria for nanoparticles used in wound healing: those that act as delivery vehicles; in the repair process; and antimicrobial activity. Due to these properties, NPs of metals such as gold, silver, copper, and zinc represent ideal candidates for application in the wound bed as well as for integration into dressings [14].

## 2. Gold Nanoparticles (GNPs)

Due to their focused delivery, safety, and increased uptake, gold nanoparticles are frequently employed for the administration of diverse bioactive compounds, enhancing drug efficiency. They are recognized as efficient transporters, and the more widely disseminated they are, the more effective their therapeutic function [15].

Due to their antioxidant and anti-inflammatory properties, these nanoparticles are utilized in the treatment of several disorders, including tissue repair [16,17]. They have substantial antioxidant properties in quenching free radicals such as OH (hydroxyl), H_2_O_2_ (hydrogen peroxide), and NO (nitric oxide) [18,19,20], depending on the surface [21]. Furthermore, for the motive that spherical gold nanoparticles have a vast surface area, they have a high proclivity to receive electrons and interact with (reactive oxygen species) ROS to remove or deactivate them [22], and so they consequently become a potent antioxidant agent and are crucial for wound healing [23]. They have strong catalytic activity in free radical scavenging processes and can also increase levels of NRF2, a factor that causes antioxidant gene activation. Keap1’s conformation is altered by GNPs’ effects on its thiol linkages, which frees NRF2 to continue the transcription of cytoprotective genes [24,25,26].

It has been observed that the topical application of GNPs, in rats, significantly accelerates the healing processes, performing a significant increase in the expression of collagen, and VEGF, in addition to cytokines (IL10 and IL4) and growth factors (FGF and TGFβ). wound closure four times faster than in the other groups [27,28]. In another study, Akturk et al., (2016) [29] evaluated its structural and morphological properties, in vitro biocompatibility, and in vivo effects such as induction or inhibition of inflammatory responses, influencing wound closure and its possible contribution to increased re-epithelialization, neovascularization, and granulation tissue formation. It was observed that these particles are associated with the secretion of cytokines (IL-8, IL-12, VEGF, and TNF-α), showing angiogenic capacity capable of increasing fibroblast proliferation and decreasing cellular apoptosis [30].

Despite their positive effects on the treatment of wounds, studies have already shown that the potential toxicity of GNPs is closely related to the dose, size, concentration, and time of exposure. GNPs have the potential to interact with the biological system and cause a natural imbalance between oxidative stress and antioxidant defense indices, which in turn can lead to various pathological effects [31,32]. The smaller its size, the greater the interaction area and the greater toxicity. In the study by Muller et al., (2017) [33] the authors demonstrated that GNPs with a size of 20 nm, at a concentration of 2.5 mg/mL for 21 days, showed potential therapeutic benefits without toxicity.

Another issue that should be taken into account is the reducing agent of the NPs. GNPs reduced with citrate tend to cause greater tissue toxicity [34]. Therefore, there was a need to study ways of synthesizing metallic NPs that would reduce their cytotoxic effects. Aloe vera leaves, *Citrullus colocynthis*, *Ocimum sanctum*, *Cinnamomum camphora*, and curcumin are just a few examples of plant extracts that have been employed to create metallic nanoparticles recently [9,35,36]. This form of synthesis guarantees a significant reduction of toxicity in biological tissues, ensuring greater safety and applicability [37].

In albino Wistar rats, gold nanoparticles produced from diverse medicinal herbs displayed extraordinary wound healing capability as well as antibacterial, antioxidant, and anti-inflammatory activity [38,39]. Aloe vera preparations containing gold and silver nanoparticles have been shown to help reduce wound infections [40,41]. The use of curcumin-based GNPs is another method that has been studied. Its biological efficacy has already been evaluated in studies with tumor cells and interaction with anticancer drugs [42], and cardiovascular studies, revealing that cardiac protection by Cur-GNPs is more effective than curcumin alone [43]. Likewise, the use of silver nanoparticles (AgNPs) stabilized with curcumin appears to be an effective strategy for wound care, as, in fact, silver nitrates have been safely used for the treatment of many ophthalmic and dental conditions and diseases, apart from wound healing.

## 3. Silver Nanoparticles (AgNPs)

Due to their low cost, chemical stability, high conductivity, catalytic activity, and broad-spectrum resistance to many pathogens, AgNPs play an important role in wound healing [26,44]. Silver has long been known as a highly antibacterial metal [45], with great efficiency towards multidrug-resistant microorganisms and biofilm-producing bacteria typically observed in chronic wounds. Size, shape, dosage, and stabilizer are all factors that influence antibacterial activity [46,47,48]. In general, the antibacterial activity of AgNPs increases dramatically as the particle size is reduced [49,50,51].

Bacterial growth can be inhibited by AgNPs through mechanisms such as the destruction of the bacterial cell membrane, and through the generation of free radicals, which induce the bacterial cell to release lipopolysaccharides and membrane proteins, resulting in cell death [52,53]. Disruption of the mitochondrial respiratory chain by AgNPs increases ROS generation and stops ATP synthesis, resulting in DNA damage [54,55]. Silver ion oxidation interactions involving oxygen and hydrogen atoms of thiol groups form disulfide bonds, which interfere with DNA replication and impede bacterial development, resulting in cell death [56,57,58]. AgNPs can also induce cell apoptosis by regulating the expression of genes such as p53 [59,60]. AgNPs are thus a viable option for applying novel biological procedures such as catheter modification, dental application, wound healing, and bone repair.

Despite being particularly powerful against a variety of germs, silver’s application is restricted due to the related tissue toxicity. AgNPs induce dose-, size- and time-dependent cytotoxicity, particularly for those with sizes ≤10 nm [46,61,62]. AgNPs of smaller sizes and large surface area tend to accumulate in mouse organs such as the liver, spleen, kidney, and brain, following intravenous, intraperitoneal, and intratracheal administration routes [63,64,65], unlike topical applications that tend to be less toxic to these organs [66]. AgNPs can eliminate microorganisms, but induce cytotoxicity in mammalian cells. In that respect, green synthesis is a safer alternative for the production of AgNPs, which use less harmful reagents from renewable sources as a reducing and stabilizing agent [67]. AuNPs coated via green synthesis have been observed to be even smaller in size, which exposes reactive and catalytic sites to rapid counterattacks during inflammation, proliferation, and remodeling [68].

The healing time of the damaged area depends on the size, dose, and morphology of the silver nanoparticles. Recently, human skin fibroblasts and keratinocytes treated with collagen-coated AgNPs encapsulated in collagen hydrogels demonstrated encouraging safety and efficacy results, maintaining their antimicrobial activity against *S. aureus*, *E. coli*, *P. aeruginosa*, and *Staphylococcus epidermis* [69]. Additionally, a higher quantity of VEGF mRNA found in keratinocytes around the edge of the wound suggested that AgNPs might aid in wound healing by promoting angiogenesis and controlling inflammatory cytokines [56].

In skin biopsies, AgNPs can persist in the cytoplasm of fibroblasts and encourage dermal and epidermis regeneration [56,70], induce keratinocyte proliferation and migration and promote fibroblast differentiation in myofibroblasts, which may aid adhesion, contraction, and early wound closure [51,70]. In addition, they regulate the production of inflammatory cytokines and proteins, such as VEGF and matrix metalloproteinases (MMPs) [30,71], which highlights the positive role of AgNPs in wound repair for the clinical treatment of wounds and postoperative results.

Metal nanoparticles can be used in gel form [72] or incorporated into scaffolds [73] to treat wounds, as a new strategy. AgNPs have properties that make them promising candidates for applications in dressings, hydrogels, and tissue engineering, which can be cited: greater mechanical stability and resistance to enzymatic degradation when incorporated into tissue structures [74,75], easy incorporation of antibodies [76], growth factors [77], peptides [78], biocompatibility [79,80], anti-inflammatory effects [81], and mainly antimicrobial properties [82,83,84].

## 4. Copper Nanoparticles (CuNPs)

The major problem of chronic wounds is due to infections and vascular problems, and just like gold and silver nanoparticles, CuNPs also have antimicrobial activities, mainly by reducing the fungal load at the wound site and increasing the healing process [85,86]. Copper was recognized in 2008 by the United States Environmental Protection Agency (EPA) as the first metallic antimicrobial agent [87]. This metal serves as a cofactor for enzymes such as superoxide dismutase and cytochrome oxidase and enhances immunity by stimulating the production of interleukin-2 [88]. Copper-based nanoparticles become engaged in all stages of wound healing because they have a complex role in numerous cells besides influencing multiple cytokine and growth factor methods of action [89].

It is a necessary metal that is needed at low levels in various metabolic activities. Copper, in fact, stimulates the synthesis of ECM components such as fibrinogen under regulated settings, stimulates the activity of MMPs in fibroblasts, and contributes to the formation of collagen and integrins, the main mediators of cellular binding to the extracellular matrix [90,91]. However, excessive copper consumption is harmful because it produces free radicals, which can cause lipid peroxidation and cell death [92,93]. The Fenton-type reaction, which produces ROS in the vicinity of copper ions and causes lipid and protein damage, has been implicated as the cause of copper toxicity mammalian cells, on the other hand, are somewhat protected by cytoplasmic metallothioneins, glutathione, and Cu/Zn superoxide dismutase [94,95]. Other studies have shown that low concentrations of copper do not cause adverse reactions when applied to human skin [96].

CuNPs have been used to speed the recovery process in animal models by inducing VEGF and angiogenesis [97,98,99] via factor-1-alpha generated by hypoxia (HIF-1), where CuNP enhances HIF-1 expression [100]. HIF-, a copper-induced auxiliary factor [101], plays a crucial role in the healing of wounds by assisting those whose peripheral blood supply is compromised (for instance, those with vascular diseases or diabetes), which prevents wounds from healing properly due to low copper levels at the wound site [102].

Several case reports have shown that dressings and hydrogels containing CuNPs provide wound protection due to their antimicrobial and fungicidal activity, in addition to stimulating the tissue repair process of the most varied types of wounds [103,104,105].

## 5. Zinc Oxide Nanoparticles (ZnONPs)

Other nanoparticles with promising characteristics for healing are those of zinc oxide (ZnONPs). These NPs are biocompatible, permeable to the dermis and epidermis, and have exhibited remarkable regenerative abilities in vivo (rat model) through re-epithelialization, keratinocyte migration along with collagen fiber deposition, and tissue granulation [106]. MMPs, a class of zinc-dependent proteins, are crucial in wound healing, as their ability to enzymatically break down collagen fragments is enhanced by the application of zinc oxide [107]. A series of experiments in which the rate of surgical wound repair was examined in rats with induced or hereditary zinc deficiency demonstrated that rats that received supplemental zinc had better surgical wound repair compared to those with zinc deficiency [108].

Modification of ZnO NPs with chitosan (CS) increased their average size, and reduced their aggregation tendency and cytotoxicity without impairing their unique properties. ZnONPs increased the degree of porosity, hydrophilicity and water absorption, oxygen permeability, and biodegradability of scaffolds. The preparation of ZnONPs scaffolds exhibits antimicrobial activity against Gram-negative and Gram-positive bacteria in the presence and absence of UV light [109]. Overall, zinc-based NPs associated with biofilms, amniotic membranes, growth factors, and collagen show great promise for applications in chronic wounds.

One of the main mechanisms of action of ZnONPs is the small increase in the production of ROS (especially H_2_O_2_), which stimulates the migration and proliferation of fibroblasts [110,111], in addition to being the main reason for the antibacterial activity of ZnONPs [112]. When the particle size decreases, the number of ZnO atoms on the surface increases, increasing H_2_O_2_ generation, which causes greater antibacterial activity.

As with other NPs, the toxicity of ZnONPs is dose-, size-, and concentration-dependent [113]. Numerous studies have demonstrated that ZnONPs are disintegrated in the external environment in the form of Zn^2+^ ions and subsequently taken up by the cell through passive dissemination over the plasma membrane, which is the critical step for cellular toxicity [114,115]. In contrast, as the particle size increases, the compatibility of ZnO nanoparticles with fibroblastic cells also increases. In the study by Kaushik Kaushik, Niranjan, Thangam, Madhan, Pandiyarasan, Ramachandran, Oh and Venkatasubbu [112], cell proliferation analysis (MTT) confirms that ZnONPs are not toxic to HDF cells. These cells showed a higher proliferation rate in the presence of larger-sized ZnONPs (55 nm) when compared to smaller-sized ZnONPs (15 nm), do not cause toxicity, and do not inhibit fibroblastic cell proliferation [112]. When in ideal doses and size, ZnONPs demonstrated anti-inflammatory and antioxidant properties [116]. ZnO nanoparticles are highly compatible with fibroblast cells and enhance the growth of these cells, promoting cell adhesion and migration [112].

In addition to the metallic NPs presented in this perspective, new NPs are already being studied in the biomedical field. Nanoparticles made of iron oxide and cobalt ferrite (CoFe_2_O_4_) have gained a lot of attention in the last decade due to their elemental properties, which make them non-toxic and biodegradable. CoFe_2_O_4_ NPs are being increasingly modified in terms of synthesis methods and nanocomposites to improve their biocompatibility and avoid potential toxicity [117].

Table 1 states the main functions and applications of metallic nanoparticles used for wound healing.

## 6. Perspectives

Given all the above, it is evident that the use of these metallic NPs is capable of developing a new therapeutic modality in the treatment of wounds, demonstrating potent effects in reducing infections caused by microorganisms and in reducing healing time, reducing the damage caused by the inflammatory process chronic. The key benefits of employing NPs include the ability to deliver medicines, genes, and peptides at elevated amounts with very few adverse effects as compared to conventional drug delivery methods, increasing the efficacy of the therapy [120,121,122]. The binding of metallic NPs with biological molecules, such as hyaluronic acid (HA), optimizes the secretion of anti-inflammatory cytokines, proliferation, and growth factors of cell differentiation and makes an earlier transition to the chronic phase, contributing to the process of repair [28,123].

Peptides and proteins have played an important role in many biological processes, functioning as enzymes, hormones, ligands, receptors, cell mediators, and structural components of cells. As intrinsic molecules in signaling pathways, peptides allow for therapeutic interventions that closely mimic natural signaling cascades. However, the short chain of amino acids in free peptides is susceptible to proteolysis in vivo. As a strategy for its use, a perspective is employed in the conjugation of peptides to metallic NPs to extend the half-life of the peptides, preventing proteolytic enzymes from degrading them [124,125].

Tissue engineering is an area on the rise, and metallic NPs can act as nanocarriers, releasing bioactive molecules in a precise and sustainable way. Nanomaterials have a high surface area to volume ratio, which allows greater penetration into the wound area to provide sustained and controlled release of therapeutic agents [126]. NPs can be integrated with various hydrogel materials such as alginate, gelatin, gelatin methacrylate, and chitosan [106,127,128]. There are several approaches to integrating NPs into hydrogels, including hydrogel formation on a preformed nanoparticle suspension or gelation of the hydrogel followed by physical incorporation of the NPs within it [129,130]. As well as the incorporation of collagen nanofibers with metallic NPs, in vivo studies already demonstrate that the healing rate of wounds treated with collagen nanofibers and metallic NPs was accelerated compared to simple collagen nanofibers, with accelerated re-epithelialization, collagen production and better wound contraction [131,132].

In addition to these hydrogels, a perspective on the use of metallic NPs with decellularized natural matrices is employed. Decellularized natural matrices (such as amniotic membranes and umbilical cords) are promising materials for tissue engineering due to their biochemical content, micro- and nanotopography, and presence of signaling molecules and growth factors [106,133]. These scaffolds can also be processed from the same patient to reduce immunogenicity. Several studies demonstrate the wide range of applications of NPs to improve structurally and functionally decellularized matrices [134,135,136]. GNPs, for example, can bind to matrices through electrostatic or covalent interactions, depending on the chemistry of the surface of the particles [136].

Stem cells are key players in the fields of tissue engineering and regenerative medicine due to their ability to differentiate into different cell lineages and types. It has already been demonstrated that conductive hybrid materials with inorganic nanostructures increase the proliferation and differentiation of adhered cells. In particular, NPs have been widely employed to produce hybrid materials to support stem cell differentiation due to their inertness, ease of surface modifications, and ability to transmit electrical signals [137,138].

Several drugs, growth factors, genes, and even the nanoparticles themselves can play different roles in tissue recovery. Furthermore, as an enhancement to scaffolds, nanoparticles can increase cell proliferation and growth factors, contributing to the regenerative healing process. The constant release of growth factors through a delivery system based on nanomaterials is an important strategy for tissue engineering [139]. These wound healing nanoscale delivery systems have better delivery of poorly water-soluble drugs, protect the therapeutic against temperature, light, pH, or enzyme degradation at the site of administration, and further stimulate fibroblast proliferation, reduce inflammation and reduce cytotoxicity of certain drugs [140]. Therefore, the involvement of nanotechnology through the therapeutically active dressing in the delivery of growth factors and pro-angiogenesis compounds along with other therapeutic agents (e.g., antimicrobials) serves as a potential revolution in wound care [141].

Furthermore, we envision that AuNPs can be used as multimodal tools, in which they not only enhance the properties of scaffolds to enhance tissue formation but also act as nanosensors. In this way, they can provide feedback on tissue function and at the same time allow for the controlled release of drugs within the dressings.

Another perspective of the use of metallic NPs is those produced through plant extracts, a method known as green synthesis, which guarantees less toxicity of these particles in biological tissues, and greater safety of applicability, in addition to adding the effects of NPs with the effects of extracts. In this way, it is suggested that these hydrogels are capable of acting as an important adjuvant with the multidisciplinary team in the treatment of wounds, accelerating the healing process, thus reducing complications such as infections, necrosis of tissues adjacent to the wounds, and also amputations of segments or even entire members. In this way, it also manages to have an economic impact by reducing costs with dressings, medicines, consultations, and other procedures, in public and private health institutions.

## Figures and Tables

**Table 1:** Main functions and applications of metallic nanoparticles used for wound healing.

Metallic Nanoparticles	Main Functions/Applications	Safe Dose and Concentration for Topical Application	References
**GNPs**	Improves efficiency of drugs, molecules (AH), and peptides /excellent carriers/anti-inflammatory and antioxidant capacity/antimicrobial action/scaffolds	≅20 nm ≤5.0 mL/L	[33] [66] [118]
**AgNPs**	Antimicrobial/cell adhesion/cell proliferation/anti-inflammatory effects	≥10 nm 1.0 mg/mL–3 mg/mL	[63] [64] [65] [66] [112]
**CuNPs**	Antimicrobial/antifungal/improves property of MMPs in fibroblasts/angiogenesis	40 nm–80 nm ≤2 mL/mL	[66] [112]
**ZnONPs**	Biocompatibility/antimicrobial/proliferation of fibroblasts and keratinocytes/deposition of collagen fibers	15 nm–55 nm 1.0 mg/mL–10 mg/mL	[112] [119] [108] [112]

## References

[1] Kazemzadeh-Narbat M., Annabi N., Khademhosseini A. (2015). Surgical sealants and high strength adhesives. Mater. Today.

[2] Morais G.F.d.C., Oliveira S.H.d.S., Soares M.J.G.O. (2008). Avaliação de feridas pelos enfermeiros de instituições hospitalares da rede pública. Texto Contexto-Enferm..

[3] Troxler M., Vowden K., Vowden P. (2006). Integrating adjunctive therapy into practice: The importance of recognising ‘hard-to-heal’wounds. World Wide Wounds.

[4] Medina A., Scott P.G., Ghahary A., Tredget E.E. (2005). Pathophysiology of Chronic Nonhealing Wounds. J. Burn. Care Rehabil..

[5] Trengove N.J., Stacey M.C., MacAuley S., Bennett N., Gibson J., Burslem F., Murphy G., Schultz G. (1999). Analysis of the acute and chronic wound environments: The role of proteases and their inhibitors. Wound Repair Regen..

[6] Harding K.G., Morris H.L., Patel G.K. (2002). Science, medicine, and the future: Healing chronic wounds. BMJ.

[7] Lauer G., Sollberg S., Cole M., Krieg T., Eming S.A., Flamme I., Stürzebecher J., Mann K. (2000). Expression and Proteolysis of Vascular Endothelial Growth Factor is Increased in Chronic Wounds. J. Investig. Dermatol..

[8] Yager D.R., Chen S.M., Ward S.I., Olutoye O.O., Diegelmann R.F., Cohen I.K. (1997). Ability of chronic wound fluids to degrade peptide growth factors is associated with increased levels of elastase activity and diminished levels of proteinase inhibitors. Wound Repair Regen..

[9] Ovais M., Ahmad I., Khalil A.T., Mukherjee S., Javed R., Ayaz M., Raza A., Shinwari Z.K. (2018). Wound healing applications of biogenic colloidal silver and gold nanoparticles: Recent trends and future prospects. Appl. Microbiol. Biotechnol..

[10] Kalashnikova I., Das S., Seal S. (2015). Nanomaterials for wound healing: Scope and advancement. Nanomedicine.

[11] Barroso A., Mestre H., Ascenso A., Simões S., Reis C. (2020). Nanomaterials in wound healing: From material sciences to wound healing applications. Nano Sel..

[12] Bhattacharya R., Mukherjee P. (2008). Biological properties of “naked” metal nanoparticles. Adv. Drug Deliv. Rev..

[13] Arvizo R.R., Bhattacharyya S., Kudgus R.A., Giri K., Bhattacharya R., Mukherjee P. (2012). Intrinsic therapeutic applications of noble metal nanoparticles: Past, present and future. Chem. Soc. Rev..

[14] Negut I., Grumezescu V., Grumezescu A.M. (2018). Treatment Strategies for Infected Wounds. Molecules.

[15] Ghosh P., Han G., De M., Kim C.K., Rotello V.M. (2008). Gold nanoparticles in delivery applications. Adv. Drug Deliv. Rev..

[16] Victor E.G., Silveira P.C., Possato J.C., da Rosa G.L., Munari U.B., de Souza C.T., A Pinho R., da Silva L., Streck E.L., Paula M.M. (2012). Pulsed ultrasound associated with gold nanoparticle gel reduces oxidative stress parameters and expression of pro-inflammatory molecules in an animal model of muscle injury. J. Nanobiotechnol..

[17] Li X., Wang H., Rong H., Li W., Luo Y., Tian K., Quan D., Wang Y., Jiang L. (2015). Effect of composite SiO 2 @AuNPs on wound healing: In vitro and vivo studies. J. Colloid Interface Sci..

[18] Esumi K., Houdatsu H., Yoshimura T. (2004). Antioxidant action by gold-PAMAM dendrimer nanocomposites. Langmuir.

[19] Leu J.-G., Chen S.-A., Chen H.-M., Wu W.-M., Hung C.-F., Yao Y.-D., Tu C.-S., Liang Y.-J. (2012). The effects of gold nanoparticles in wound healing with antioxidant epigallocatechin gallate and α-lipoic acid. Nanomedicine.

[20] Medhe S., Bansal P., Srivastava M.M. (2014). Enhanced antioxidant activity of gold nanoparticle embedded 3,6-dihydroxyflavone: A combinational study. Appl. Nanosci..

[21] Yakimovich N.O., Ezhevskii A.A., Guseinov D.V., Smirnova L.A., Gracheva T.A., Klychkov K.S. (2008). Antioxidant properties of gold nanoparticles studied by ESR spectroscopy. Bull. Acad. Sci. USSR Div. Chem. Sci..

[22] BarathManiKanth S., Kalishwaralal K., Sriram M., Pandian S.R.K., Youn H.-S., Eom S., Gurunathan S. (2010). Anti-oxidant effect of gold nanoparticles restrains hyperglycemic conditions in diabetic mice. J. Nanobiotechnol..

[23] Muthuvel A., Adavallan K., Balamurugan K., Krishnakumar N. (2014). Biosynthesis of gold nanoparticles using Solanum nigrum leaf extract and screening their free radical scavenging and antibacterial properties. Biomed. Prev. Nutr..

[24] Rattanata N., Daduang S., Wongwattanakul M., Leelayuwat C., Limpaiboon T., Lekphrom R., Sandee A., Boonsiri P., Chio-Srichan S., Daduang J. (2015). Gold Nanoparticles Enhance the Anticancer Activity of Gallic Acid against Cholangiocarcinoma Cell Lines. Asian Pac. J. Cancer Prev..

[25] Cheng H., Lai G., Fu L., Zhang H., Yu A. (2015). Enzymatically catalytic deposition of gold nanoparticles by glucose oxidase-functionalized gold nanoprobe for ultrasensitive electrochemical immunoassay. Biosens. Bioelectron..

[26] Wang X., Chang J., Wu C. (2018). Bioactive inorganic/organic nanocomposites for wound healing. Appl. Mater. Today.

[27] Kim J.E., Lee J., Jang M., Kwak M.H., Go J., Kho E.K., Song S.H., Sung J.E., Lee J., Hwang D.Y. (2015). Accelerated healing of cutaneous wounds using phytochemically stabilized gold nanoparticle deposited hydrocolloid membranes. Biomater. Sci..

[28] Mendes C., Haupenthal D.P.D.S., Zaccaron R.P., Silveira G.D.B., Corrêa M.E.A.B., Casagrande L.D.R., Mariano S.D.S., Silva J.I.D.S., de Andrade T.A.M., Feuser P.E. (2020). Effects of the Association between Photobiomodulation and Hyaluronic Acid Linked Gold Nanoparticles in Wound Healing. ACS Biomater. Sci. Eng..

[29] Akturk O., Kismet K., Yasti A.C., Kuru S., E Duymus M., Kaya F., Caydere M., Hucumenoglu S., Keskin D. (2016). Collagen/gold nanoparticle nanocomposites: A potential skin wound healing biomaterial. J. Biomater. Appl..

[30] Franková J., Pivodová V., Vágnerová H., Juránová J., Ulrichova J. (2016). Effects of silver nanoparticles on primary cell cultures of fibroblasts and keratinocytes in a wound-healing model. J. Appl. Biomater. Funct. Mater..

[31] Abdelhalim M.A.A.-K., Al-Ayed M.S., Moussa S. (2015). The effects of intraperitoneal administration of gold nanoparticles size and exposure duration on oxidative and antioxidants levels in various rat organs. Pak. J. Pharm. Sci..

[32] Ferreira G.K., Cardoso E., Vuolo F.S., Michels M., Zanoni E.T., Carvalho-Silva M., Gomes L.M., Dal-Pizzol F., Rezin G.T., Streck E.L. (2015). Gold nanoparticles alter parameters of oxidative stress and energy metabolism in organs of adult rats. Biochem. Cell Biol..

[33] Muller A.P., Ferreira G.K., Pires A.J., de Bem Silveira G., de Souza D.L., de Abreu Brandolfi J., de Souza C.T., Paula M.M., Silveira P.C.L. (2017). Gold nanoparticles prevent cognitive deficits, oxidative stress and inflammation in a rat model of sporadic dementia of Alzheimer’s type. Mater. Sci. Eng..

[34] Rónavári A., Igaz N., Adamecz D.I., Szerencsés B., Molnar C., Kónya Z., Pfeiffer I., Kiricsi M. (2021). Green Silver and Gold Nanoparticles: Biological Synthesis Approaches and Potentials for Biomedical Applications. Molecules.

[35] Gupta A., Briffa S.M., Swingler S., Gibson H., Kannappan V., Adamus G., Kowalczuk M.M., Martin C., Radecka I. (2020). Synthesis of Silver Nanoparticles Using Curcumin-Cyclodextrins Loaded into Bacterial Cellulose-Based Hydrogels for Wound Dressing Applications. Biomacromolecules.

[36] Mittal A.K., Bhaumik J., Kumar S., Banerjee U.C. (2014). Biosynthesis of silver nanoparticles: Elucidation of prospective mechanism and therapeutic potential. J. Colloid Interface Sci..

[37] Zhang X.-F., Liu Z.-G., Shen W., Gurunathan S. (2016). Silver Nanoparticles: Synthesis, Characterization, Properties, Applications, and Therapeutic Approaches. Int. J. Mol. Sci..

[38] Awan U.A., Ali S., Rehman M., Zia N., Naz S.S., Ovais M., Raza A. (2018). Stable and reproducible synthesis of gold nanorods for biomedical applications: A comprehensive study. IET Nanobiotechnol..

[39] Milaneze B., Keijok W., Jairo O., Brunelli P., Janine B., Larissa L., Adilson P., Denise E., Maria P., Moises R. (2014). The green synthesis of gold nanoparticle using extract of Virola oleifera. BMC Proc..

[40] Chandran S.P., Chaudhary M., Pasricha R., Ahmad A., Sastry M.J.B. (2006). Synthesis of gold nanotriangles and silver nanoparticles using Aloevera plant extract. Biotechnol. Prog..

[41] Naraginti S., Kumari P.L., Das R.K., Sivakumar A., Patil S.H., Andhalkar V.V. (2016). Amelioration of excision wounds by topical application of green synthesized, formulated silver and gold nanoparticles in albino Wistar rats. Mater. Sci. Eng. C.

[42] Waghmare M.N., Qureshi T.S., Krishna C.M., Pansare K., Gadewal N., Hole A., Dongre P.M. (2021). β-Lactoglobulin-gold nanoparticles interface and its interaction with some anticancer drugs—An approach for targeted drug delivery. J. Biomol. Struct. Dyn..

[43] Sharifiaghdam Z., Dalouchi F., Sharifiaghdam M., Shaabani E., Ramezani F., Nikbakht F., Azizi Y. (2022). Curcumin-coated gold nanoparticles attenuate doxorubicin-induced cardiotoxicity via regulating apoptosis in a mouse model. Clin. Exp. Pharmacol. Physiol..

[44] Yah C.S., Simate G.S.J.D.J.o.P.S. (2015). Nanoparticles as potential new generation broad spectrum antimicrobial agents. DARU J. Pharm. Sci..

[45] Konop M., Damps T., Misicka A., Rudnicka L. (2016). Certain Aspects of Silver and Silver Nanoparticles in Wound Care: A Minireview. J. Nanomater..

[46] Jeong Y., Lim D.W., Choi J. (2014). Assessment of Size-Dependent Antimicrobial and Cytotoxic Properties of Silver Nanoparticles. Adv. Mater. Sci. Eng..

[47] Burkowska-But A., Sionkowski G., Walczak M. (2014). Influence of stabilizers on the antimicrobial properties of silver nanoparticles introduced into natural water. J. Environ. Sci..

[48] Oei J.D., Zhao W.W., Chu L., DeSilva M.N., Ghimire A., Rawls H.R., Whang K. (2012). Antimicrobial acrylic materials with in situ generated silver nanoparticles. J. Biomed. Mater. Res. Part B Appl. Biomater..

[49] Raza M.A., Kanwal Z., Rauf A., Sabri A.N., Riaz S., Naseem S. (2016). Size- and Shape-Dependent Antibacterial Studies of Silver Nanoparticles Synthesized by Wet Chemical Routes. Nanomaterials.

[50] Agnihotri S., Mukherji S., Mukherji S.J.R.A. (2014). Size-controlled silver nanoparticles synthesized over the range 5–100 nm using the same protocol and their antibacterial efficacy. Rsc Adv..

[51] Gunasekaran T., Nigusse T., Dhanaraju M.D. (2011). Silver Nanoparticles as Real Topical Bullets for Wound Healing. J. Am. Coll. Clin. Wound Spéc..

[52] Mijakovic I., Petranovic D., Bottini N., Deutscher J., Jensen P.R. (2005). Protein-Tyrosine Phosphorylation in Bacillus subtilis. Microb. Physiol..

[53] Shrivastava S., Bera T., Roy A., Singh G., Ramachandrarao P., Dash D. (2007). Characterization of enhanced antibacterial effects of novel silver nanoparticles. Nanotechnology.

[54] Grzelak A., Wojewódzka M., Męczyńska-Wielgosz S., Zuberek M., Wojciechowska D., Kruszewski M. (2018). Crucial role of chelatable iron in silver nanoparticles induced DNA damage and cytotoxicity. Redox Biol..

[55] AshaRani P., Hande M.P., Valiyaveettil S. (2009). Anti-proliferative activity of silver nanoparticles. BMC Cell Biol..

[56] Rigo C., Ferroni L., Tocco I., Roman M., Munivrana I., Gardin C., Cairns W.R.L., Vindigni V., Azzena B., Barbante C. (2013). Active Silver Nanoparticles for Wound Healing. Int. J. Mol. Sci..

[57] Vijayakumar V., Samal S.K., Mohanty S., Nayak S.K. (2019). Recent advancements in biopolymer and metal nanoparticle-based materials in diabetic wound healing management. Int. J. Biol. Macromol..

[58] Singla R., Soni S., Patial V., Kulurkar P.M., Kumari A., Mahesh S., Padwad Y.S., Yadav S.K. (2017). Cytocompatible Anti-microbial Dressings of Syzygium cumini Cellulose Nanocrystals Decorated with Silver Nanoparticles Accelerate Acute and Diabetic Wound Healing. Sci. Rep..

[59] Wang Z., Chen C., Wang Y., Li F., Huang J., Luo Z., Rao S., Tan Y., Liu Y., Yin H. (2019). Ångstrom-Scale Silver Particles as a Promising Agent for Low-Toxicity Broad-Spectrum Potent Anticancer Therapy. Adv. Funct. Mater..

[60] Farah M.A., Ali M.A., Chen S.-M., Li Y., Al-Hemaid F.M., Abou-Tarboush F.M., Al-Anazi K.M., Lee J. (2016). Silver nanoparticles synthesized from Adenium obesum leaf extract induced DNA damage, apoptosis and autophagy via generation of reactive oxygen species. Colloids Surf. B Biointerfaces.

[61] Butler K.S., Peeler D.J., Casey B.J., Dair B.J., Elespuru R.K. (2015). Silver nanoparticles: Correlating nanoparticle size and cellular uptake with genotoxicity. Mutagenesis.

[62] Dayem A.A., Hossain M.K., Lee S.B., Kim K., Saha S.K., Yang G.-M., Choi H.Y., Cho S.-G. (2017). The Role of Reactive Oxygen Species (ROS) in the Biological Activities of Metallic Nanoparticles. Int. J. Mol. Sci..

[63] Jiang X., Lu C., Tang M., Yang Z., Jia W., Ma Y., Jia P., Pei D., Wang H. (2018). Nanotoxicity of Silver Nanoparticles on HEK293T Cells: A Combined Study Using Biomechanical and Biological Techniques. ACS Omega.

[64] Trickler W.J., Lantz S.M., Murdock R.C., Schrand A.M., Robinson B.L., Newport G.D., Schlager J.J., Oldenburg S.J., Paule M.G., Slikker W.J.T.S. (2010). Silver nanoparticle induced blood-brain barrier inflammation and increased permeability in primary rat brain microvessel endothelial cells. Toxicol. Sci..

[65] Sokołowska P., Białkowska K., Siatkowska M., Rosowski M., Kucińska M., Komorowski P., Makowski K., Walkowiak B. (2017). Human brain endothelial barrier cells are distinctly less vulnerable to silver nanoparticles toxicity than human blood vessel cells: A cell-specific mechanism of the brain barrier?. Nanomed. Nanotechnol. Biol. Med..

[66] Xu C., Akakuru O.U., Ma X., Zheng J., Zheng J., Wu A. (2020). Nanoparticle-Based Wound Dressing: Recent Progress in the Detection and Therapy of Bacterial Infections. Bioconjug. Chem..

[67] Montaser A., Abdel-Mohsen A., Ramadan M., Sleem A., Sahffie N., Jancar J., Hebeish A. (2016). Preparation and characterization of alginate/silver/nicotinamide nanocomposites for treating diabetic wounds. Int. J. Biol. Macromol..

[68] Tian J., Wong K.K., Ho C.M., Lok C.N., Yu W.Y., Che C.M., Cliu J.F., Tam P.K. (2007). Topical delivery of silver nanoparticles promotes wound healing. ChemMedChem.

[69] Das S., Baker A.B. (2016). Biomaterials and Nanotherapeutics for Enhancing Skin Wound Healing. Front. Bioeng. Biotechnol..

[70] Liu X., Lee P.-Y., Ho C.-M., Lui V.C.H., Chen Y., Che C.-M., Tam P.K.H., Wong K.K.Y. (2010). Silver Nanoparticles Mediate Differential Responses in Keratinocytes and Fibroblasts during Skin Wound Healing. ChemMedChem.

[71] Chowdhury S., De M., Guha R., Batabyal S., Samanta I., Hazra S.K., Ghosh T.K., Konar A., Hazra S. (2014). Influence of silver nanoparticles on post-surgical wound healing following topical application. Eur. J. Nanomed..

[72] Silveira P.C., Venâncio M., Souza P.S., Victor E.G., Notoya F.D.S., Paganini C.S., Streck E.L., da Silva L., Pinho R.A., Paula M.M. (2014). Iontophoresis with gold nanoparticles improves mitochondrial activity and oxidative stress markers of burn wounds. Mater. Sci. Eng. C.

[73] Cozad M.J., Bachman S.L., Grant S.A. (2011). Assessment of decellularized porcine diaphragm conjugated with gold nanomaterials as a tissue scaffold for wound healing. J. Biomed. Mater. Res. Part A.

[74] Grant S.A., Spradling C.S., Grant D.N., Fox D.B., Jimenez L., Grant D.A., Rone R.J. (2014). Assessment of the biocompatibility and stability of a gold nanoparticle collagen bioscaffold. J. Biomed. Mater. Res. Part A.

[75] Deeken C.R., Fox D.B., Bachman S.L., Ramshaw B.J., Grant S.A. (2011). Characterization of bionanocomposite scaffolds comprised of amine-functionalized gold nanoparticles and silicon carbide nanowires crosslinked to an acellular porcine tendon. J. Biomed. Mater. Res. Part B Appl. Biomater..

[76] Obaid G., Chambrier I., Cook M.J., Russell D.A. (2015). Cancer targeting with biomolecules: A comparative study of photodynamic therapy efficacy using antibody or lectin conjugated phthalocyanine-PEG gold nanoparticles. Photochem. Photobiol. Sci..

[77] Paviolo C., Chon J.W.M., Clayton A.H.A. (2015). Inhibiting EGFR Clustering and Cell Proliferation with Gold Nanoparticles. Small.

[78] Gopalakrishnan R., Singam E.R.A., Sundar J.V., Subramanian V. (2015). Interaction of collagen like peptides with gold nanosurfaces: A molecular dynamics investigation. Phys. Chem. Chem. Phys..

[79] Cui Q., Yashchenok A., Zhang L., Li L., Masic A., Wienskol G., Möhwald H., Bargheer M. (2014). Fabrication of Bifunctional Gold/Gelatin Hybrid Nanocomposites and Their Application. ACS Appl. Mater. Interfaces.

[80] Hung H.-S., Chang C.-H., Chang C.-J., Tang C.-M., Kao W.-C., Lin S.-Z., Hsieh H.-H., Chu M.-Y., Sun W.-S., Hsu S.-H. (2014). In Vitro Study of a Novel Nanogold-Collagen Composite to Enhance the Mesenchymal Stem Cell Behavior for Vascular Regeneration. PLoS ONE.

[81] Chen H., Dorrigan A., Saad S., Hare D.J., Cortie M.B., Valenzuela S.M. (2013). In Vivo Study of Spherical Gold Nanoparticles: Inflammatory Effects and Distribution in Mice. PLoS ONE.

[82] Regiel-Futyra A., Kus-Liśkiewicz M., Sebastian V., Irusta S., Arruebo M., Stochel G., Kyzioł A. (2015). Development of Noncytotoxic Chitosan–Gold Nanocomposites as Efficient Antibacterial Materials. ACS Appl. Mater. Interfaces.

[83] Ehmann H.M., Breitwieser D., Winter S., Gspan C., Koraimann G., Maver U., Sega M., Köstler S., Stana-Kleinschek K., Spirk S. (2015). Gold nanoparticles in the engineering of antibacterial and anticoagulant surfaces. Carbohydr. Polym..

[84] Li X., Robinson S.M., Gupta A., Saha K., Jiang Z., Moyano D.F., Sahar A., Riley M.A., Rotello V.M. (2014). Functional Gold Nanoparticles as Potent Antimicrobial Agents against Multi-Drug-Resistant Bacteria. ACS Nano.

[85] El-Abeid S.E., Ahmed Y., Daròs J.-A., Mohamed M.A. (2020). Reduced Graphene Oxide Nanosheet-Decorated Copper Oxide Nanoparticles: A Potent Antifungal Nanocomposite against Fusarium Root Rot and Wilt Diseases of Tomato and Pepper Plants. Nanomaterials.

[86] Shende S., Bhagat R., Raut R., Rai M., Gade A. (2021). Myco-Fabrication of Copper Nanoparticles and Its Effect on Crop Pathogenic Fungi. IEEE Trans. NanoBiosci..

[87] Vincent M., Duval R.E., Hartemann P., Engels-Deutsch M. (2018). Contact killing and antimicrobial properties of copper. J. Appl. Microbiol..

[88] Hopkins R.G., Failla M.L. (1997). Copper Deficiency Reduces Interleukin-2 (IL-2) Production and IL-2 mRNA in Human T-Lymphocytes. J. Nutr..

[89] Kornblatt A.P., Nicoletti V.G., Travaglia A. (2016). The neglected role of copper ions in wound healing. J. Inorg. Biochem..

[90] Borkow G., Gabbay J., Dardik R., Eidelman A.I., Lavie Y., Grunfeld Y., Ikher S., Huszar M., Zatcoff R.C., Marikovsky M. (2010). Molecular mechanisms of enhanced wound healing by copper oxide-impregnated dressings. Wound Repair Regen..

[91] Uauy R., Olivares M., Gonzalez M. (1998). Essentiality of copper in humans. Am. J. Clin. Nutr..

[92] Quaranta D., Krans T., Santo C.E., Elowsky C.G., Domaille D.W., Chang C.J., Grass G. (2011). Mechanisms of Contact-Mediated Killing of Yeast Cells on Dry Metallic Copper Surfaces. Appl. Environ. Microbiol..

[93] Palza H. (2015). Antimicrobial Polymers with Metal Nanoparticles. Int. J. Mol. Sci..

[94] Hatori Y., Clasen S., Hasan N.M., Barry A., Lutsenko S. (2012). Functional Partnership of the Copper Export Machinery and Glutathione Balance in Human Cells. J. Biol. Chem..

[95] Babula P., Masarik M., Adam V., Eckschlager T., Stiborova M., Trnkova L., Skutkova H., Provaznik I., Hubalek J., Kizek R. (2012). Mammalian metallothioneins: Properties and functions. Metallomics.

[96] Hostynek J.J., Maibach H.I. (2003). Copper Hypersensitivity: Dermatologie Aspects. Rev. Environ. Health.

[97] Das A., Sudhahar V., Chen G.-F., Kim H.W., Youn S.-W., Finney L., Vogt S., Yang J., Kweon J., Surenkhuu B. (2016). Endothelial Antioxidant-1: A Key Mediator of Copper-dependent Wound Healing in vivo. Sci. Rep..

[98] Alizadeh S., Seyedalipour B., Shafieyan S., Kheime A., Mohammadi P., Aghdami N. (2019). Copper nanoparticles promote rapid wound healing in acute full thickness defect via acceleration of skin cell migration, proliferation, and neovascularization. Biochem. Biophys. Res. Commun..

[99] Chen M., Li R., Yin W., Wang T., Kang Y.J. (2020). Copper promotes migration of adipose-derived stem cells by enhancing vimentin-Ser39 phosphorylation. Exp. Cell Res..

[100] Abdollahi Z., Zare E., Salimi F., Goudarzi I., Tay F., Makvandi P. (2021). Bioactive Carboxymethyl Starch-Based Hydrogels Decorated with CuO Nanoparticles: Antioxidant and Antimicrobial Properties and Accelerated Wound Healing In Vivo. Int. J. Mol. Sci..

[101] Ruthenborg R.J., Ban J.-J., Wazir A., Takeda N., Kim A.J.-W. (2014). Regulation of Wound Healing and Fibrosis by Hypoxia and Hypoxia-Inducible Factor-1. Mol. Cells.

[102] Borkow G., Zatcoff R.C., Gabbay J. (2009). Reducing the risk of skin pathologies in diabetics by using copper impregnated socks. Med. Hypotheses.

[103] Baek J.H., Yoo M.A., Koh J.S., Borkow G. (2012). Reduction of facial wrinkles depth by sleeping on copper oxide-containing pillowcases: A double blind, placebo controlled, parallel, randomized clinical study. J. Cosmet. Dermatol..

[104] Dykes P. (2015). Increase in skin surface elasticity in normal volunteer subjects following the use of copper oxide impregnated socks. Ski. Res. Technol..

[105] Melamed E., Kiambi P., Okoth D., Honigber I., Tamir E., Borkow G. (2021). Healing of Chronic Wounds by Copper Oxide-Impregnated Wound Dressings—Case Series. Medicina.

[106] Yadid M., Feiner R., Dvir T. (2019). Gold Nanoparticle-Integrated Scaffolds for Tissue Engineering and Regenerative Medicine. Nano Lett..

[107] Khalid A., Khan R., Ul-Islam M., Khan T., Wahid F. (2017). Bacterial cellulose-zinc oxide nanocomposites as a novel dressing system for burn wounds. Carbohydr. Polym..

[108] Kogan S., Sood A., Garnick M.S. (2017). Zinc and Wound Healing: A Review of Zinc Physiology and Clinical Applications. Wounds.

[109] Díez-Pascual A.M., Díez-Vicente A.L.J.B. (2015). Wound healing bionanocomposites based on castor oil polymeric films reinforced with chitosan-modified ZnO nanoparticles. Biomacromolecules.

[110] Sharma D.K., Sharma K.K., Kumar V., Sharma A. (2016). Effect of Ce doping on the structural, optical and magnetic properties of ZnO nanoparticles. J. Mater. Sci. Mater. Electron..

[111] Augustine R., Dominic E.A., Reju I., Kaimal B., Kalarikkal N., Thomas S. (2014). Investigation of angiogenesis and its mechanism using zinc oxide nanoparticle-loaded electrospun tissue engineering scaffolds. RSC Adv..

[112] Kaushik M., Niranjan R., Thangam R., Madhan B., Pandiyarasan V., Ramachandran C., Oh D.-H., Venkatasubbu G.D. (2019). Investigations on the antimicrobial activity and wound healing potential of ZnO nanoparticles. Appl. Surf. Sci..

[113] Czyżowska A., Barbasz A. (2022). A review: Zinc oxide nanoparticles–friends or enemies?. Int. J. Environ. Health Res..

[114] Osmond M.J., Mccall M.J. (2010). Zinc oxide nanoparticles in modern sunscreens: An analysis of potential exposure and hazard. Nanotoxicology.

[115] Muller E.B., Hanna S.K., Lenihan H.S., Miller R.J., Nisbet R.M. (2014). Impact of engineered zinc oxide nanoparticles on the energy budgets of Mytilus galloprovincialis. J. Sea Res..

[116] Manuja A., Raguvaran R., Kumar B., Kalia A., Tripathi B. (2020). Accelerated healing of full thickness excised skin wound in rabbits using single application of alginate/acacia based nanocomposites of ZnO nanoparticles. Int. J. Biol. Macromol..

[117] Barani M., Rahdar A., Mukhtar M., Razzaq S., Qindeel M., Olam S.A.H., Paiva-Santos A.C., Ajalli N., Sargazi S., Balakrishnan D. (2022). Recent application of cobalt ferrite nanoparticles as a theranostic agent. Mater. Today Chem..

[118] Kushwaha A., Goswami L., Kim B.S. (2022). Nanomaterial-Based Therapy for Wound Healing. Nanomaterials.

[119] Melnikova N., Balakireva A., Orekhov D., Kamorin D., Didenko N., Malygina D., Knyazev A., Novopoltsev D., Solovyeva A. (2021). Zinc Oxide Nanoparticles Protected with Terpenoids as a Substance in Redox Imbalance Normalization in Burns. Pharmaceuticals.

[120] Singh R., Pantarotto D., McCarthy D., Chaloin O., Hoebeke J., Partidos C.D., Briand J.-P., Prato M., Bianco A., Kostarelos K. (2005). Binding and Condensation of Plasmid DNA onto Functionalized Carbon Nanotubes: Toward the Construction of Nanotube-Based Gene Delivery Vectors. J. Am. Chem. Soc..

[121] Bruchez M., Moronne M., Gin P., Weiss S., Alivisatos A.P. (1998). Semiconductor Nanocrystals as Fluorescent Biological Labels. Science.

[122] Wang S., Mamedova N., Kotov N.A., Chen W., Studer J. (2002). Antigen/Antibody Immunocomplex from CdTe Nanoparticle Bioconjugates. Nano Lett..

[123] El-Aassar M.R., Ibrahim O.M., Fouda M.M.G., El-Beheri N.G., Agwa M.M. (2020). Wound healing of nanofiber comprising Polygalacturonic/Hyaluronic acid embedded silver nanoparticles: In-vitro and in-vivo studies. Carbohydr. Polym..

[124] Moretta R., Terracciano M., Rea I., Stefano L.D. (2021). Bioconjugation of Peptides to Hybrid Gold Nanoparticles. Peptide Conjugation.

[125] Maraming P., Kah J.C.Y. (2021). Conjugation of peptides to gold nanoparticles. Peptide Conjugation.

[126] Hamdan S., Pastar I., Drakulich S., Dikici E., Tomic-Canic M., Deo S., Daunert S. (2017). Nanotechnology-Driven Therapeutic Interventions in Wound Healing: Potential Uses and Applications. ACS Central Sci..

[127] Yoo J., Lee E., Kim H.Y., Young K.H., Jung J., Kim H., Chang Y., Lee W., Shin J., Baek S. (2017). Electromagnetized gold nanoparticles mediate direct lineage reprogramming into induced dopamine neurons in vivo for Parkinson’s disease therapy. Nat. Nanotechnol..

[128] Baei P., Jalili-Firoozinezhad S., Rajabi-Zeleti S., Tafazzoli-Shadpour M., Baharvand H., Aghdami N. (2016). Electrically conductive gold nanoparticle-chitosan thermosensitive hydrogels for cardiac tissue engineering. Mater. Sci. Eng. C.

[129] Lu J., Chen Y., Ding M., Fan X., Hu J., Chen Y., Li J., Li Z., Liu W. (2022). A 4arm-PEG macromolecule crosslinked chitosan hydrogels as antibacterial wound dressing. Carbohydr. Polym..

[130] Lai W.-F., Wong W.-T. (2021). Property-Tuneable Microgels Fabricated by Using Flow-Focusing Microfluidic Geometry for Bioactive Agent Delivery. Pharmaceutics.

[131] Rath G., Hussain T., Chauhan G., Garg T., Goyal A.K. (2016). Collagen nanofiber containing silver nanoparticles for improved wound-healing applications. J. Drug Target..

[132] Ragothaman M., Villalan A.K., Dhanasekaran A., Palanisamy T. (2021). Bio-hybrid hydrogel comprising collagen-capped silver nanoparticles and melatonin for accelerated tissue regeneration in skin defects. Mater. Sci. Eng. C.

[133] Corrêa M.E.A.B., Mendes C., Bittencourt J.V.S., Takejima A., de Souza I.C., de Carvalho S.C.D., Orlandini I.G., de Andrade T.A.M., Guarita-Souza L.C., Silveira P.C.L. (2022). Effects of the Application of Decellularized Amniotic Membrane Solubilized with Hyaluronic Acid on Wound Healing. Ann. Biomed. Eng..

[134] Londono R., Badylak S.F. (2015). Biologic Scaffolds for Regenerative Medicine: Mechanisms of In vivo Remodeling. Ann. Biomed. Eng..

[135] Edri R., Gal I., Noor N., Harel T., Fleischer S., Adadi N., Green O., Shabat D., Heller L., Shapira A. (2019). Personalized Hydrogels for Engineering Diverse Fully Autologous Tissue Implants. Adv. Mater..

[136] Wali N., Shabbir A., Wajid N., Abbas N., Naqvi S.Z.H. (2022). Synergistic efficacy of colistin and silver nanoparticles impregnated human amniotic membrane in a burn wound infected rat model. Sci. Rep..

[137] Baranes K., Shevach M., Shefi O., Dvir T. (2016). Gold Nanoparticle-Decorated Scaffolds Promote Neuronal Differentiation and Maturation. Nano Lett..

[138] Heo D.N., Castro N., Lee S.-J., Noh H., Zhu W., Zhang L.G. (2017). Enhanced bone tissue regeneration using a 3D printed microstructure incorporated with a hybrid nano hydrogel. Nanoscale.

[139] Alavi M., Rai M. (2020). Topical delivery of growth factors and metal/metal oxide nanoparticles to infected wounds by polymeric nanoparticles: An overview. Expert Rev. Anti-Infect. Ther..

[140] Alberti T., Coelho D.S., Voytena A., Pitz H., De Pra M., Mazzarino L., Kuhnen S., Ribeiro-Do-Valle R.M., Maraschin M., Veleirinho B. (2017). Nanotechnology: A Promising Tool Towards Wound Healing. Curr. Pharm. Des..

[141] Seifalian A.M., Naderi N., Karponis D., Mosahebi A. (2018). Nanoparticles in wound healing from hope to promise from promise to routine. Front. Biosci..

